# Gametophyte Thermal Priming Modifies the Transcriptomic Heat Stress Response of Sporophyte Progeny in *Saccharina latissima*

**DOI:** 10.1111/eva.70301

**Published:** 2026-07-23

**Authors:** Anne M. L. Nilsen, Niko Steiner, Inka Bartsch, J. Mark Cock, Alexander O. Jueterbock

**Affiliations:** ^1^ Faculty of Biosciences and Aquaculture Nord University Bodø Norway; ^2^ Alfred Wegener Institute Helmholtz‐Centre for Polar‐ and Marine Research Bremerhaven Germany; ^3^ Algal Genetics Group, CNRS Sorbonne University, UMR 8227 Roscoff France

**Keywords:** kelp, priming, *Saccharina latissima*, stress memory, thermal tolerance, transcriptomics

## Abstract

*Saccharina latissima* (sugar kelp) is a brown macroalga that forms kelp forests along North Atlantic coasts, playing a key role in coastal ecosystems. It is also of growing economic importance as the dominant species used in European kelp aquaculture. Both wild and cultivated kelps are increasingly threatened by marine heatwaves and elevated sea surface temperatures. Enhancing thermal tolerance could therefore benefit both conservation and production. Thermal priming, a method originating in agriculture where early life stages are exposed to moderate heat stress, can enhance resilience by inducing molecular stress memory. We applied a thermal priming treatment (20°C, 3 weeks) to *S. latissima* gametophytes before gametogenesis and sporophyte formation to test whether priming alters thermal responses in the derived sporophytes. Young sporophytes, produced by crossing primed or naïve (controls kept at 10°C) gametophytes, were reared at 10°C and then exposed to a heat stress (21.5°C for 48 h) before being allowed to recover at 10°C. Transcriptomic profiles and photophysiological characteristics were assessed before, during and after stress exposure to investigate priming‐induced changes in thermal stress resilience. Sporophytes derived from primed gametophytes showed stronger transcriptomic responses under heat stress; for example, upregulation of HSP90B was unique to primed samples. Naïve sporophytes exhibited a delayed response with extensive downregulation during recovery (2027 DEGs). Gene ontology enrichment analysis indicated higher expression of protein phosphorylation, chloroplast‐related functions, and post‐transcriptional regulation by ncRNA in primed sporophytes, suggesting a priming effect on their transcriptomic thermal response. Primed sporophytes exhibited a delayed reduction in photosynthetic performance compared with naïve throughout the heat stress. These findings suggest that thermal priming induces a transgenerational stress memory. Our results support the view that temperature priming alters the transcriptomic thermal response in kelp through regulatory changes in gene expression, in line with existing literature on priming.

**Abbreviations**
ABS: absorption flux by the PSII antenna
ANOVA: analysis of variance
BP: biological process
CC: cellular component
DEG: differentially expressed gene
F_0_
: minimal chlorophyll fluorescence yield
F_m_
: maximal chlorophyll fluorescence yield
*F*_v_/*F*_m_
: maximum quantum yield of photosystem II
GO: gene ontology
HSP: heat shock protein
HT: high temperature (sampling time point in our experiment, day 4)
LFC: Log2 fold change
ncRNA: non‐coding RNA
OJIP: fluorescence transient O‐J‐I‐P (with steps: Origin, J‐step, I‐step, and Peak)
PAM: pulse‐amplitude modulated (fluorometer)
PCA: principal component analysis
PES: provasoli‐enriched seawater
*PI*_*abs*_
: performance index (on absorption basis)
PSII: photosystem II
QA: the primary quinone electron acceptor in PSII
QTL: quantitative trait locus
R: recovery (sampling time point in our experiment, day 7)
RC: active photosystem II reaction center
RNA‐seq: RNA sequencing
ROS: reactive oxygen species
S: start (pre‐stress sampling time point in our experiment, day 1)

## 1 Introduction

Kelps, large brown macroalgae of the order Laminariales, are marine primary producers that form extensive forests, providing food and habitat for diverse marine organisms such as fish and invertebrates (Dayton 1985; Smale et al. 2013; Teagle et al. 2017). They also deliver key ecosystem services such as water quality improvement, coastal erosion protection, and nutrient cycling (Kosek and Kukliński 2023; Smale et al. 2013). *Saccharina latissima* (Linnaeus) Lane et al. (2006), or sugar kelp, is an ecologically and economically important kelp species in the Northern Hemisphere, and is widely studied due to its broad distribution and economic interest (Diehl et al. 2024).

Kelp forests are in decline globally due to ocean warming, marine heatwaves, pollution, and sea urchin grazing (de Bettignies et al. 2021; Filbee‐Dexter and Scheibling 2014; Filbee‐Dexter and Wernberg 2018; Smale 2020). For instance, sugar kelp forest is a red‐listed ecosystem type in Norway, and significant decline in kelp forests have been documented across the North Atlantic (de Bettignies et al. 2021; Gundersen et al. 2018). Declining kelp forests are often replaced with turf algae, which suppress kelp recruitment and forest recovery, representing an ecological tipping point (Farrell et al. 2025; Filbee‐Dexter and Wernberg 2018; Schiel et al. 2018). Restoration methods, such as the use of green gravel, where kelp gametophytes or spores are seeded onto gravel for dispersion, have been proposed to counteract these losses (Chemello et al. 2024; Fredriksen et al. 2020).

Beyond their ecological significance, kelps are also important aquaculture organisms. They are cultivated for use in food (e.g., kombu), animal feed, biostimulants, and cosmetics (Sæther et al. 2024; Slegers et al. 2021). Interest in macroalgae cultivation, which is widespread in Asia (FAO 2020), is growing across Europe and the Americas (Araújo et al. 2021; Grebe et al. 2019). The emerging aquaculture has stimulated scientific interest in cultivation techniques and strain improvement, particularly for *Saccharina latissima* and *Alaria esculenta* , the most widely farmed species in the North Atlantic (Bråtelund et al. 2025; Ebbing et al. 2021; Huang et al. 2023; Nehr et al. 2025). Selective breeding applied in Asian aquaculture has effectively contributed to producing high yields and improving product quality (FAO 2020; Hu et al. 2021; Zhang et al. 2022). However, the establishment of kelp cultivars in Europe is limited by regulatory restrictions and concerns regarding spread into wild populations (farm to wild gene flow) and reduced pathogen resistance due to reduced genetic diversity in selectively bred strains (Goecke et al. 2020).

An alternative or complementary strategy to breeding is priming, a pre‐treatment that enhances resilience or growth by inducing molecular stress memory (Hilker et al. 2016; Jueterbock et al. 2021; Martinez‐Medina et al. 2016). Priming has been widely applied in agriculture to improve resistance to abiotic or biotic stress or to accelerate germination or growth. Treatments such as chemical or microbial exposure, or temperature shifts are applied to early life stages, for example plant seeds, or in the case of kelps, gametophytes. These treatments can establish molecular memories through mechanisms such as DNA methylation, histone modifications, or non‐coding RNAs (Hilker et al. 2016; Iwasaki and Paszkowski 2014; Jueterbock et al. 2021). These epigenetic marks can be partially heritable, enabling the priming effect to persist for one or several generations (Hilker et al. 2016; Iwasaki and Paszkowski 2014; Jueterbock et al. 2021). Although most research on priming has focused on terrestrial plants, recent studies demonstrate that thermal priming can enhance heat stress tolerance in macroalgae such as *Laminaria digitata* , *S. latissima*, and the red alga *Bangia* sp. (Gauci et al. 2024; Kishimoto et al. 2019; Sato et al. 2024).

Priming, which enables organisms to better respond to abiotic stressors, induces changes in transcriptomic profiles. In bacteria and yeasts, gene expression profiles associated with improved stress responses are induced by prior exposures to related stimuli (Hilker et al. 2016). In plants, priming can establish both short‐ and long‐term transcriptome changes. Some genes maintain altered expression after priming, while other genes revert to their earlier expression levels but respond more quickly or strongly to repeated stress exposure (Harris et al. 2023). These transcriptome effects are associated with changes in epigenetic marks, with short‐term priming memories linked to chromatin restructuring around promoters or methylation marks associated with actively transcribed or repressed genes, while long‐term transgenerational memories may result from DNA methylation repressing genes or silencing transposable elements (Harris et al. 2023). Whether gene expression is positively or negatively correlated with methylation depends on several contexts, such as the position of the methylation within genes or regulatory regions or on tissue type (Liu et al. 2023; Luo et al. 2018). In land plants, repeated stress exposures may also “train” stress‐responsive genes to show differential expression in response to stress (Avramova 2015; Hilker and Schmülling 2019). Thus, priming enables earlier, stronger, or more effective transcriptomic responses to abiotic or biotic stress, mediated by epigenetic marks such as DNA methylation or histone post‐translational modifications (Harris et al. 2023; Hilker et al. 2016; Hilker and Schmülling 2019; Jueterbock et al. 2021).

Kelp transcriptomes, including that of *S. latissima*, display widespread transcriptomic changes under heat stress, especially affecting redox regulation, photosynthesis, and protein synthesis. Differential expression analyses in *S. latissima* have shown that kelps exposed to heat stress typically downregulate photosynthetic and other homeostasis‐related genes (Heinrich et al. 2012; Nehr et al. 2025). These patterns are in agreement with findings from other brown algae, where heat stress commonly represses transcription of photosynthesis‐related genes, though in some cases photosynthesis genes are upregulated, as in *Saccharina japonica* (Dittami et al. 2009; Hara et al. 2022; Liu et al. 2014). Kelps also consistently upregulate genes encoding heat‐shock proteins under heat stress (Dittami et al. 2009; Hara et al. 2022; Heinrich et al. 2012; Liu et al. 2014). These transcriptomic responses are examples of the most commonly observed stress responses to heat stress in brown algae.

*Saccharina latissima* and other kelps have diplohaplontic life cycles, alternating between haploid gametophytes and diploid sporophytes. Epigenetic marks established by treatments applied to gametophytes must be preserved after fertilization of eggs by spermatozoids, and thus transition from the haploid to the diploid state to persist into the sporophyte generation and yield effects, an important consideration for the application of priming.

This study explores the effect of warm temperature priming on the transcriptomic and physiological response to short‐term heat stress in young sporophytes of *Saccharina latissima*. Sporophytes derived from primed compared to naïve gametophytes showed differential responses to heat stress at the transcriptome level. Therefore, the priming memory must have been maintained through fertilization, persisting from the gametophyte to the sporophyte generation. To date, no study has characterized the transcriptomic basis of thermal priming across the gametophyte‐to‐sporophyte transition in kelp. We hypothesize that thermal priming alters the transcriptomic response of sporophytes under heat stress, enabling a faster, stronger, or more efficient recovery response compared to non‐primed counterparts, and discuss the implications for kelp cultivation and restoration.

## 2 Materials and Methods

### 2.1 Temperature Priming of Gametophytes

**FIGURE 1:**
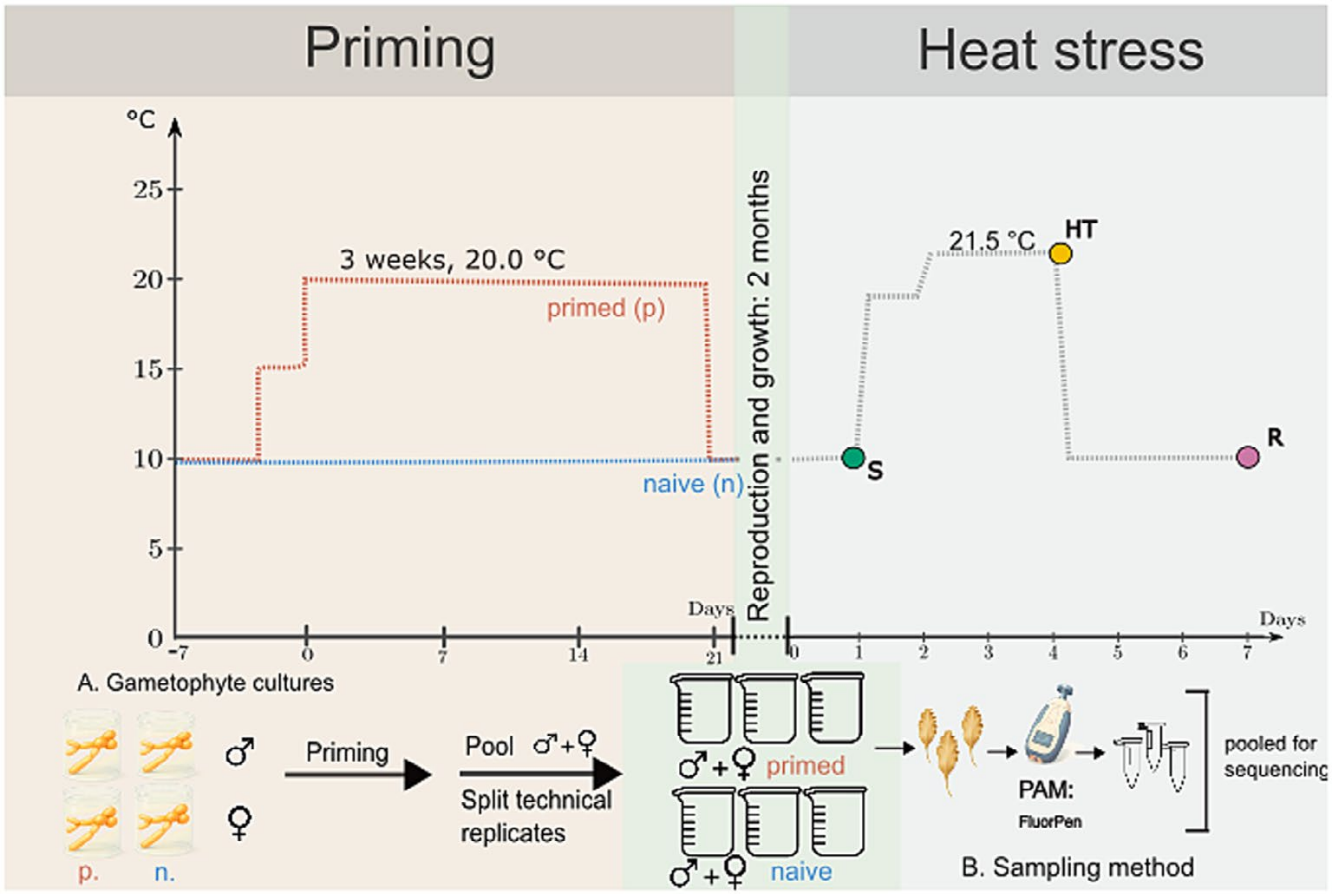
Overview of the experimental design with the priming treatment of gametophytes and the short‐term heat stress experiment performed with the resulting sporophytes. S: “Start” sampling at Day 1. HT: “High temperature” sampling at Day 4 after 24 h at 18°C followed by 48 h at 21.5°C. R: “Recovery” sampling at Day 7 after 3 days at 10.°C. A. Overview of the gametophyte cultures: Female and male gametophytes (each a combination of 7 clonal lines) were primed separately, then combined after priming into one primed and one naïve stock culture, which were then split to three technical replicates each before fertility was induced. B. Overview of sampling method: From each beaker, three sporophytes were sampled at each of the time points S, HT and R.

To identify and characterize the establishment of a transgenerational priming memory, we performed a temperature priming treatment on gametophytes. We used seven male and seven female gametophyte cultures originating from Lofoten, Norway (68°08′20.5″N 13°27′25.3″ E, collected Nov. 2022; Alfred Wegener Institute culture numbers 3640–3645, 3650–3653, and 3670–3673). The cultures were maintained at 10°C (incubators: PHCBI MIR‐154‐PE) under red light (light source: LED strip, Cotech) in autoclaved seawater enriched with iron‐free ½ strength Provasoli nutrient solution (Provasoli 1968), that is, ½ strength Provasoli‐enriched seawater (½ strength PES), to prevent gametogenesis (Bartsch 2018; Lewis et al. 2013; Lüning and Dring 1975). Vegetative gametophytes were pooled into one male and one female stock culture, which were then fragmented using ceramic mortar and pestle and evenly distributed by fresh weight into 100 mL Petri dishes for the priming treatment or the naïve control treatment (Figure 1). Both groups were maintained under 4 μmol m^−2^ s^−1^red light with a 14:10 light: dark cycle. For the warm priming treatment, we increased the temperature from 10°C to 20°C in 5°C intervals over 48 h, and then maintained a temperature of 20°C for 3 weeks before reducing the temperature back to 10°C. The naïve group was kept at 10°C. Male and female gametophytes were primed separately and combined immediately after priming. After priming, fertility was induced by switching to ½ PES medium with iron, and white light at 10 μmol m^−2^ s^−1^ with a 14:10 light: dark cycle (light source: LED strip, Cotech) (Bartsch 2018; Lewis et al. 2013; Lüning and Dring 1975). After combining, the cultures were split into three technical replicates each for the primed and naïve groups in 100 mL Petri dishes (6 in total) with weekly medium changes. Five weeks after priming, the sporophytes were placed in beakers containing 1 L ½ strength PES (Figure 1). The medium was changed weekly, and after 2 weeks standard strength PES was used (Provasoli 1968). The number of sporophytes in each beaker was reduced to 20 to maintain equal density.

### 2.2 Triggering Heat Stress Experiment

To investigate the effect of the gametophyte priming on the sporophyte's response to heat stress, we performed a heat stress experiment. Once the sporophytes reached a size of approx. 3 cm, 9 weeks after the end of priming, we increased the temperature from 10°C to 18°C for 24 h, then to 21.5°C for 48 h, followed by a 72 h recovery period at 10°C (Figure 1). Each replicate beaker contained 20 sporophytes. We sampled three sporophytes from each of the three beakers from the naïve and primed groups (in total nine primed and nine naïve sporophytes) at the experiment start (Day 1). The sampling was repeated at Day 4 after 48 h at the highest temperature 21.5°C, and at Day 7 after 3 days of recovery at 10°C (“S”, “HT” and “R” in Figure 1). To assess the effect of temperature stress on photosynthetic performance, sampled sporophytes were dark‐acclimated for 10 min at their respective treatment temperatures and measured using a clip‐on Pulse‐Amplitude Modulation (PAM) fluorometer (FluorPen FP 110, PSI, Czech Republic). The leaf clip enclosed a thallus section of approximately 10 mm diameter for dark adaptation, and exposed a spot of 5 mm diameter in the middle of the blade to the modulated measuring and saturating light pulses. The brief, localized dark adaptation and exposure to measuring and saturating light pulses may in principle induce transcriptional responses. However, as the measurement was applied identically across all samples, any transcriptional response induced by the dark acclimation would be shared across all groups and would not appear as differentially expressed genes, as it would be canceled out by the normalization procedures of the DESeq2 R package used for analysis. The maximum quantum yield of PSII ($F_{v}/F_{m}=\frac{F_{m}-F_{0}}{F_{m}}$), where *F*_0_ represents the minimal and F_m_ the maximal chlorophyll fluorescence yield, and the performance index *PI*_*abs*_ ($PI_{\mathrm{abs}}=\frac{\mathrm{RC}/\mathrm{ABS}}{1-\mathrm{RC}/\mathrm{ABS}}\cdot \frac{\varphi_{\mathrm{Po}}}{1-\varphi_{\mathrm{Po}}}\cdot \frac{\psi_{o}}{1-\psi_{o}}$) were recorded for a general indication of photosynthetic function. *PI*_*abs*_ describes PSII in more detail by integrating the density of active PSII reaction centers per absorbed light energy (RC/ABS), the maximum trapping efficiency ($\phi_{P}0$), and the efficiency of electron transport beyond the primary quinone electron acceptor QA ($\psi_{0}$), and is more sensitive to abiotic stress than *F*_v_/*F*_m_, and can therefore be beneficial for estimating stress performance in short experiments (Kalaji et al. 2014; Solovchenko et al. 2022; Strasser et al. 2000; Zuo et al. 2025). *PI*_*abs*_ is derived from the OJIP measurement (Rapid fluorescence transient measurement O‐J‐I‐P with steps: Origin, J‐step, I‐step, and Peak) (Kalaji et al. 2014). Default settings for OJIP fluorescence transient (OJIP) were used for PAM measurements, as adjustments of pulse intensity did not lead to higher measurement values. After PAM measurements the sporophytes were flash‐frozen in liquid nitrogen and stored at −80°C. This same procedure was performed at each sampling day.

To test for differences in photosynthetic performance while accounting for repeated sampling of sporophytes from the same beakers, we performed 2‐way repeated measures ANOVAs with Tukey HSD post hoc tests with studentized range adjustment for the effects of priming and time point on *F*_v_/*F*_m_ and *PI*_*abs*_, using the R environment for statistical computing v.4.4.3 with packages car v.3.1‐3 and emmeans v.1.11.2 (Fox and Weisberg 2019; Lenth 2025; R Core Team 2025; Tukey 1949; White 1980). Post hoc comparisons were conducted using estimated marginal means with the appropriate within‐subjects error terms. Because multiple sporophytes were sampled from each beaker, beaker identity was included as a random effect to account for non‐independence of individuals sampled from the same beaker. We assessed the data's compliance with the ANOVA's assumptions of normality and homogeneity of variance with Shapiro–Wilk's test and Levene's test (Levene 1960; Shapiro and Wilk 1965). As necessary, data was transformed prior to the ANOVAs using the Box‐Cox method (Box and Cox 1964), with R package MASS (Venables and Ripley 2002) to estimate the optimal λ parameters (*F*_v_/*F*_m_: 2.00, *PI*_*abs*_: 0.22), where λ determines the power needed to best approximate a normal distribution and stabilize variances (Box and Cox 1964). Sphericity was evaluated with Mauchly's test and was not violated for either response variable (all *p* > 0.05), so uncorrected repeated‐measures ANOVA results are reported (Mauchly 1940).

### 2.3 Transcriptome Sequencing and Bioinformatic Analyses

Frozen samples were sent to Novogene Co. for RNA extraction and sequencing. RNA was isolated using the RNAprep Pure Tissue Kit for Plant (TIANGEN). Tissue (0.1–0.5 g) was processed with the kit according to the manufacturer's protocol, and RNA was eluted in RNase‐free water. Purity and concentration were verified with a Nanodrop (Thermo Fisher Scientific, USA) and a Qubit fluorometer (Thermo Fisher Scientific, USA). Three sporophytes sampled at the same time from the same beaker were pooled to obtain sufficient RNA. Pooling of sporophytes should have reduced the risk of strong effects due to genotype by tending to average out genotypic effects. However, pooling is also likely to have increased variance within samples, leading to a more conservative interpretation of priming effects on transcriptomics. Strand‐specific RNA‐seq (ssRNA‐seq) libraries were prepared using an inverse rRNA depletion protocol to enrich for mRNA. Libraries were constructed as paired‐end libraries and sequenced by Novogene on an Illumina NovaSeq X Plus platform, producing 150 bp paired‐end reads. On average 105 million paired‐end reads were generated per sample (range: 68.5–137.4 million reads). Adapter trimming and quality filtering of RNA‐seq reads was performed with Trim Galore v 0.6.10 (Krueger et al. 2023), removing reads with Phred quality < 30 and performing FastQC quality control. The filtered reads were mapped onto the *Saccharina latissima* reference genome v2 assembly (Denoeud et al. 2024, https://phaeoexplorer.sb‐roscoff.fr/) using HISAT2 v. 2.2.1 (Kim et al. 2019), and gene‐level read counts were generated using featureCounts v2.0.3 (Liao et al. 2014). Differential expression analysis was performed using DESeq2 v. 1.46.0 (Love et al. 2014). To account for repeated sampling of sporophytes from the same beakers, we followed the DESeq2 vignette method for group‐specific condition effects (Love et al. 2025), with the design “~ treatment + treatment:beaker.*n* + priming:time_point”. From the DESeq2 analysis, contrasts were extracted for the effect of the high temperature (HT) and recovery (R) time points compared with the starting point separately for the primed and naïve group, as well as the differential expression between the primed and naïve group at the experiment start. In addition, the condition effect of the priming treatment was extracted to determine the difference in gene expression between the primed and naïve group at the HT and R time points. Expression counts were normalized by the built‐in normalization of DESeq2 (Love et al. 2014). Genes with an absolute log2FoldChange (LFC) of ≥ 2 and a Benjamini‐Hochberg adjusted *p*‐value of < 0.05 were considered to be significant differentially expressed genes (DEGs) (Benjamini and Hochberg 1995).

For an overview of the transcriptome differences between the sample groups, differential gene expression in the samples was illustrated with principal component analysis (PCA). Using the DEseq2 and ggpubr v. 0.6.1 R packages (Kassambara 2025; Love et al. 2014), a PCA plot was drawn with primed and naïve groups split for the time points “Start”, “High temperature” and “Recovery”. The plot was based on the 500 most variably expressed genes according to the DEG analysis.

To investigate the functional aspects of the differential gene expression, we performed gene ontology enrichment (GO) analysis. Using the output from the differential expression analysis, the GO analysis was carried out with the topGO v. 2.58.0 R package (Alexa and Rahnenfuhrer 2016), using GO terms from the *Saccharina latissima* v2.0 genome annotation (https://phaeoexplorer.sb‐roscoff.fr/) (Denoeud et al. 2024). The *S. latissima* reference genome comprises 18,169 predicted protein‐coding genes, as reported in the Phaeoexplorer database (Denoeud et al. 2024, https://phaeoexplorer.sb‐roscoff.fr/). Of these, the associated eggNOG‐mapper annotation file includes 12,223 gene models, of which 4336 have at least one assigned GO identifier (Denoeud et al. 2024, https://phaeoexplorer.sb‐roscoff.fr/). To prioritize GO terms with the most differentially expressed genes and counteract annotation bias, we used the Kolmogorov–Smirnov method (Smirnov 1939), and the DEGs were weighted by LFC using “algorithm = ‘weight01’”.

To functionally annotate *S. latissima* transcripts, we used the eggNOG‐mapper (emapper v2.1.9) and InterProScan (v5.59–91.0) annotations available from the Phaeoexplorer database (https://phaeoexplorer.sb‐roscoff.fr/). These annotation files provided descriptive annotations for 1866 of 3045 unique DEGs across all pairwise comparisons in our data.

Because only 424 of our DEGs were associated with Gene Ontology (GO) terms, we complemented the GO enrichment analysis with a manual keyword search targeting the eggNOG‐ and InterProScan‐derived descriptions. Using a list of stress‐ and physiology‐related keywords grouped into broad functional categories (full list: Data S1), we matched DEG descriptions to one or more categories and then summarized category‐specific DEG counts for each comparison to obtain an overview of transcripts relevant to heat stress responses and epigenetic memory. We chose broad functional categories that capture core processes with known relevance to heat stress responses in plants or algae. Heat shock and protein folding, oxidative stress and ROS detoxification, photosynthesis and PSII protection, lipid metabolism and membrane protection, and cell wall remodeling and growth regulation were included because these pathways modulate proteostasis, redox balance, photosynthetic performance, membrane stability, and acclimation involved in heat stress responses and temperature‐sensitive processes in plants and algae (Barati et al. 2019; Chen et al. 2026; Heinrich et al. 2012; Lin et al. 2024; Lu et al. 2025; Mathur et al. 2014). In addition, we defined an epigenetic regulation and stress memory category to capture chromatin‐ and RNA‐based mechanisms implicated in stress memory and priming. However, these annotations should be interpreted with caution, as confidence levels vary. Moreover, the directionality of differential expression within functional categories is interpreted cautiously, as up‐ or downregulation of individual genes does not necessarily directly reflect biological outcomes.

## 3 Results

### 3.1 Priming Delays Photophysiological Effects of Heat Stress

**FIGURE 2:**
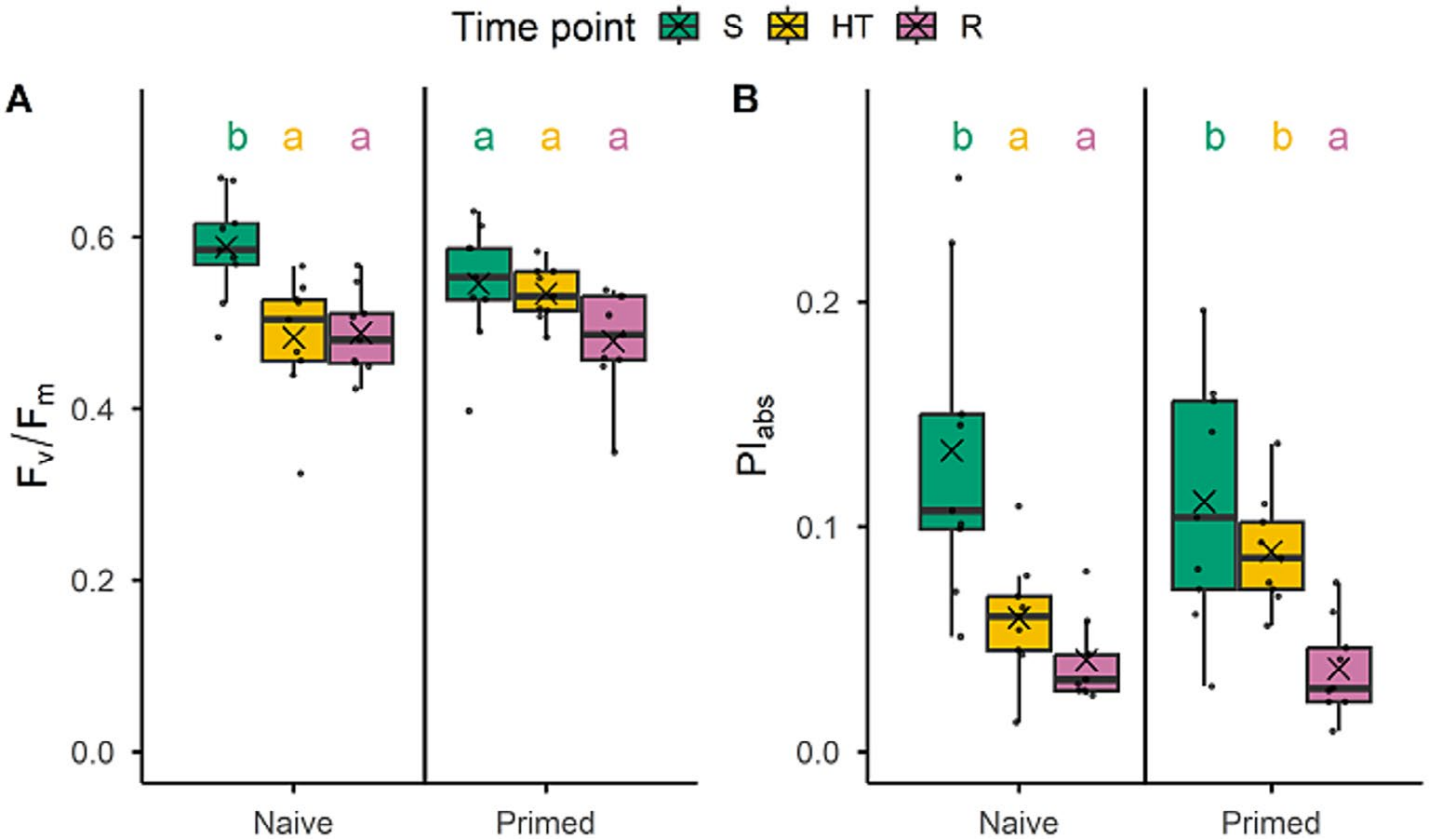
(A) Maximum quantum yield of photosystem II (*F*_*v*_/*F*_*m*_) values of the naïve and primed sporophytes sampled at different time points of the experiment: S: Start/day 1, HT: High temperature/Day 4, and R: Recovery/Day 7. (B) Performance index (*PI*_*abs*_) values of the naïve and primed sporophytes. Statistical significance (adj. *p* cutoff = 0.05) was extracted from Tukey post hoc tests from the 2‐way ANOVA and indicated with letters above the boxplots. *F*_*v*_/*F*_*m*_ and *PI*_*abs*_ values were box‐cox transformed prior to statistical tests, but the untransformed values are shown in the boxplot. Significant differences within the primed and naïve groups (not between groups) are indicated with letters “a” and “b”. The lower and upper edges of the boxed represent the 25th and 75th percentiles while the lines represent the medians and the crosses represent the means. Each box represents a total of nine sporophytes sampled from three beakers at each time point.

Primed and naive sporophytes were subjected to a heat shock and photosynthetic performance was assessed over the course of the heat shock experiment. The primed sporophytes initially showed lower *F*_*v*_ */F*_*m*_ values than naïve (although not significantly lower) under benign conditions at the experiment start (Figure 2 and Data S3). In the 2‐way repeated measures ANOVA, *PI*_*abs*_ (*p* = 0.001) values changed significantly across the time points of the experiment, indicating that both primed and naive algae were significantly impacted by the high temperature treatment. There was no significant interaction effect between treatment and time point for either of the metrics when testing for the overall effect (*F*_v_/*F*_m_: *p* = 0.107, *PI*_*abs*_: *p* = 0.179), indicating that primed and naïve samples responded similarly throughout the experiment. However, the naïve samples experience a significant decrease in *F*_v_/*F*_m_ (*p* = 0.009) and *PI*_*abs*_ (*p* = 0.026) at the HT time point compared to the S time point, in contrast to the primed samples, which showed no significant change (*F*_v_/*F*_m_: *p* = 0.830, *PI*_*abs*_: *p* = 0.821). The primed group exhibited a significant decrease in photosynthetic performance (*PI*_*abs*_) only under recovery (Figure 2 and Data S3). Thus, the primed samples showed subtly more stable *F*_v_/*F*_m_ and delayed reduction in *PI*_*abs*_ throughout the experiment.

### 3.2 Priming Alters the Transcriptomic Heat‐Stress Response

**FIGURE 3:**
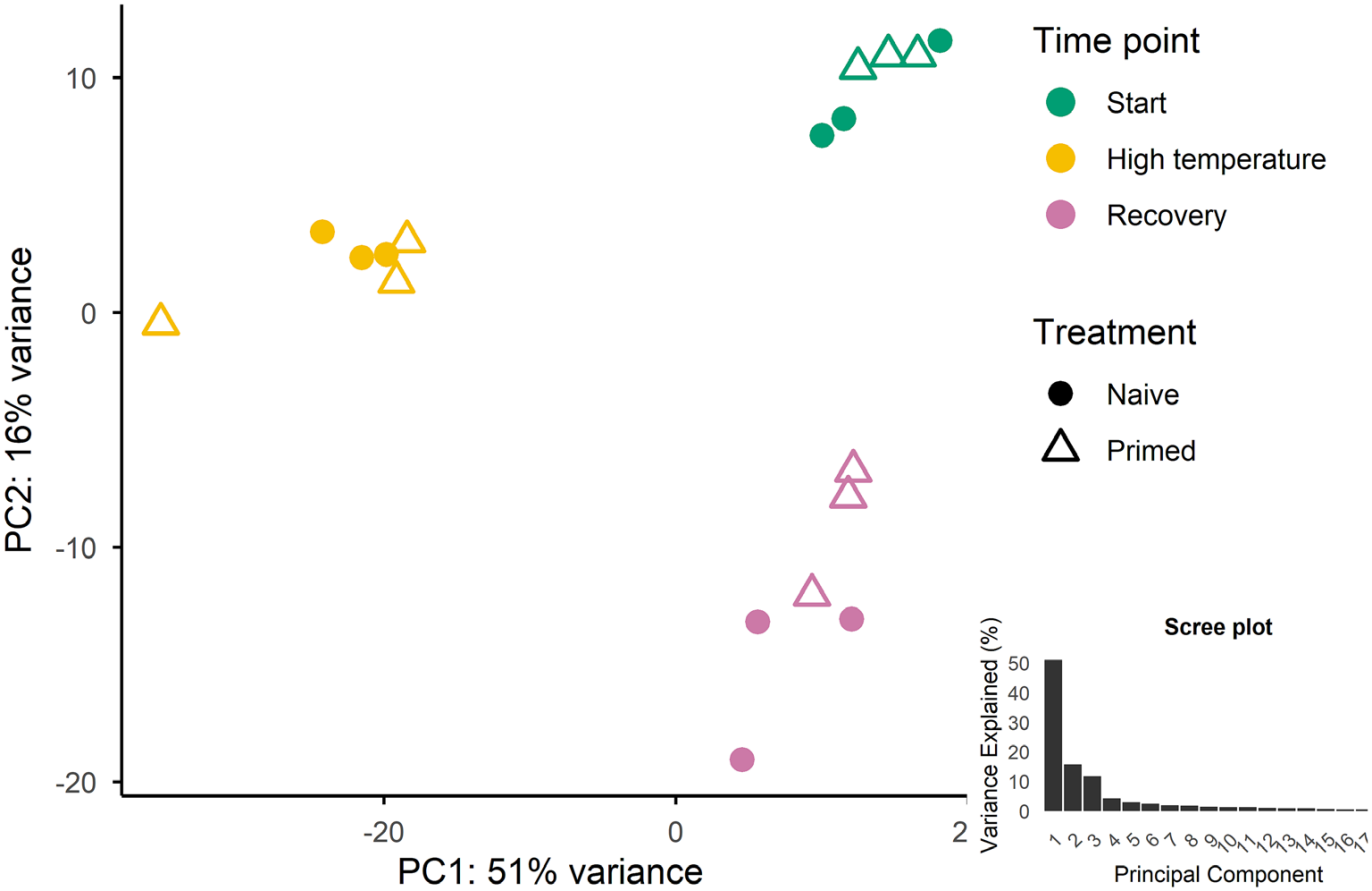
Principal component analysis (PCA) plot illustrates the gene expression of the top 500 most differentially expressed genes of naïve and primed juvenile sporophytes before, during and after the temperature treatment. Because of the pooling of the sporophytes at sequencing, the points represent the average gene expression of three sporophytes each. PC1: Principal component 1. PC2: Principal component 2.

The transcriptomes of primed and naive sporophytes were analysed over the course of the heat shock experiment using an RNA‐seq approach. A PCA plot of the 500 most differentially expressed genes across all treatment groups (Figure 3), which explained 67% of the variance in gene expression, showed that gene expression clearly differed across the S, HT and R time points, which cluster into separate groups (Figure 3).

**FIGURE 4:**
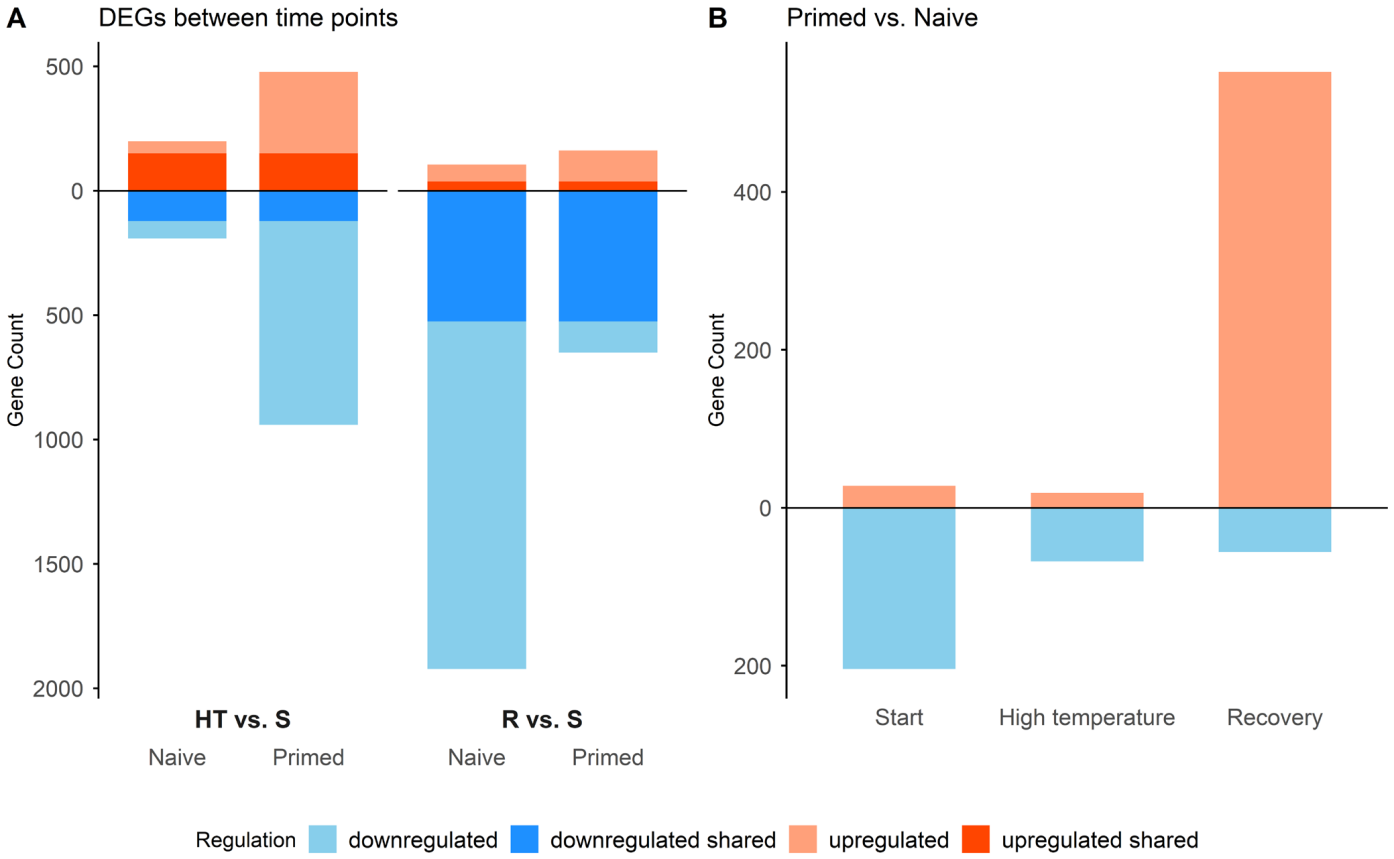
A comparison of differentially expressed genes (DEGs) counts for the groups in the heat stress triggering experiment. (A) At each time point, the gene expression of the primed and naïve group is compared, where “upregulated” means higher expression in the primed group. Sample size per group: *N* = 3 (3 pools of 3 sporophytes each). (B) At the high temperature (HT) and recovery (R) points, the gene expression of the naïve and primed samples is compared to the start (S). In these comparisons, the bolder red and blue colors indicate genes which are upregulated/downregulated in both the primed and naïve groups at the relevant time point. Total DEG counts for each comparison are printed below the bars. Sample size per group: *N* = 3 (3 pools of 3 sporophytes each).

To determine if priming alters the timing and/or intensity of the stress response at the transcriptome level, we compared the counts of differentially expressed genes (DEGs) between primed and naïve sporophytes across the three sampling time points and compared DEGs of the HT and R time points with the starting state, with the resulting gene counts shown in Data S1. The DEG analysis showed a marked temporal shift in gene regulation: primed samples showed a rapid response during the high temperature stage (HT) compared to the starting state (S) with 1419 DEGs (Figure 4A). In contrast, naïve samples showed a delayed reaction peaking only at the recovery stage (R, 3 days after returning to 10°C) compared to the starting state, with 2027 DEGs. We also observed a greater difference between primed and naïve sporophytes at the recovery stage compared to during the heat stress (Figure 4B). At recovery, the DEGs with higher expression in primed compared to naïve samples (Figure 4B) could be partially explained by a great number of downregulated DEGs in the naïve samples at recovery. Overall, the DEG analysis indicated a stronger transcriptome response in the primed samples during the heat stress, while the naïve samples show a stronger response at recovery with downregulated DEGs (Figure 4B).

### 3.3 Priming Alters the Expression of Stress‐Related Genes

The results of the GO analysis showed significantly enriched GO terms for the following groups: HT vs. S and R vs. S for both primed and naïve samples, and for primed vs. naïve at all time points (Data S2: For naïve samples, some of the top enrichments were “response to heat”, “response to chemical”, and “metabolic process” at HT, and repression of growth‐related GO terms and “cell communication” at recovery). For primed samples, some of the most significant enrichments were “peptide transport” and “lipid metabolic processes” and repression of “cytoplasm” related genes at HT (Data S2). In the comparison of primed vs. naïve samples under recovery, GO terms based on genes with higher expression in the primed samples were significantly enriched (Data S2). These GO terms, which represent functional aspects of the most differentially expressed genes between the primed and naïve groups, were GO:1900368 “regulation of post‐transcriptional gene silencing by regulatory ncRNA”, GO:0006468 “protein phosphorylation”, and GO:0009507 “chloroplast” (Data S2).

**FIGURE 5:**
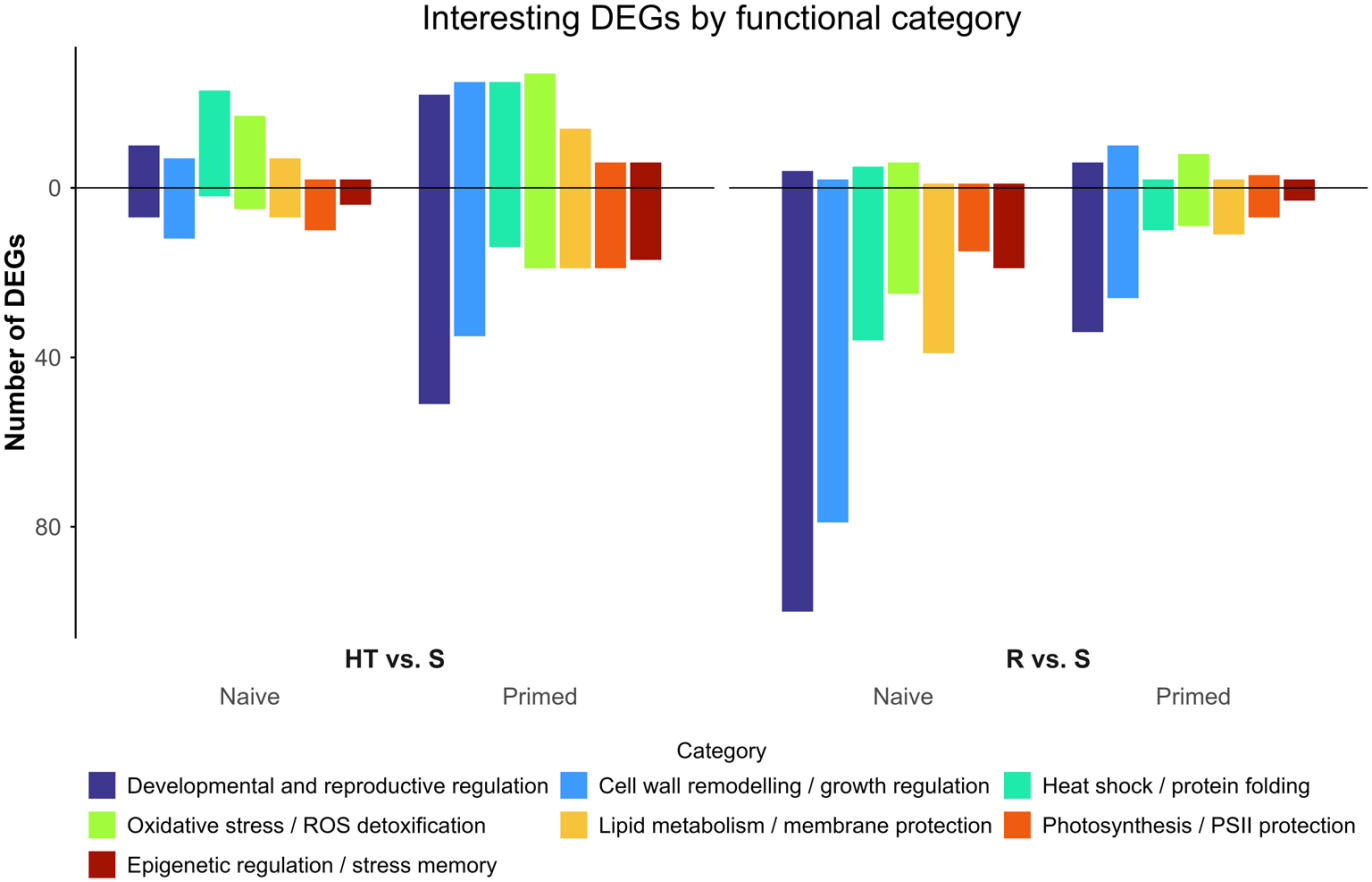
An overview of differentially expressed genes assigned to functional categories associated with heat stress responses and epigenetic regulation across naive and primed samples at the high temperature (HT) and recovery (R) time points compared with the experiment start (S). Upregulated genes are shown above and downregulated genes below the x‐axis.

The functional interpretation of stress‐relevant DEGs revealed similar patterns as the overall DEG counts: primed samples show a stronger transcriptomic response at the HT time point, with higher counts of up‐ and downregulated genes in all categories, for example “developmental and reproductive regulation” (dominated by kinases and kinase‐like domains), “heat shock/protein folding” [mostly ubiquitin and heat shock proteins (HSP), and “Oxidative stress/ROS detoxification” (containing for example thioredoxins and redox related processes)] (Figure 5 and Data S1). The DEGs in the cell wall remodeling and growth regulation category are mainly related to several pectin lyase folds. The primed samples also showed differential expression of epigenetic and stress memory related transcripts such as methyltransferases and histone‐related activity (Data S1). In contrast, naïve samples showed lower overall DEG counts in the same categories at the HT time point, but greater downregulated counts at the R time point (Figure 5). Differences in number of DEGs between functional groups are expected to reflect differences in numbers of genes annotated to each category rather than regulation differences among groups, while differences in regulation between comparisons reflect differential responses between groups.

Heat shock proteins (HSPs, including HSPs of HSP20, HSP40, HSP70, and HSP90 families) were predominantly upregulated for both groups at the HT time point with the notable exception of one strongly downregulated (LFC‐19) HSP40/DnaJ domain (SL_15_133.9) in primed samples. The same HSP40/DnaJ transcript is strongly downregulated in naive samples only at the R time point, consistent with the pattern of a later onset transcriptomic response. HSP90B is only upregulated in primed samples (SL_14_7478) at the HT time point. At the R time point, the HSP signal is reduced in primed samples with only 3 DEGs (1 upregulated, 2 downregulated), while the naive group shows 8 HSP‐related DEGs (3 upregulated, 5 downregulated). A similar pattern of mixed up‐ and downregulation is also present in several photosynthesis and redox‐related transcripts, including thioredoxin‐like proteins and ferredoxin–NADP reductases, with some transcripts such as “cprA” showing strong downregulation in primed samples at the HT time point and in naive samples only at the R time point (Data S1).

## 4 Discussion

Recent studies have demonstrated that both cold and warm priming can enhance the temperature tolerance of kelps. For example, warm priming of *Saccharina latissima* gametophytes at 20°C over a period of 4 weeks increased growth of young sporophytes by up to 30% when grown at 5°C for 21 days (Gauci et al. 2024). In the same study, priming also extended sporophyte survival at 24°C, a temperature exceeding the experimentally determined lethal limit for the species (23°C, Lüning 1984), by 4 days, with a remaining pigmented area > 5% of the initial area as a proxy for survival (Gauci et al. 2024). Warm priming of *S. latissima* gametophytes has also been shown to increase the survival of microscopic sporophytes at 21.5°C for 1 week, but the effect did not last for a second week (Steiner et al. 2026). In addition, gametophyte warm priming slightly improved re‐growth in juvenile sporophytes following tissue loss after heat exposure (Steiner et al. 2026). Cold treatments of kelp (*Laminaria digitata*) gametophytes at 5°C improved the growth of the following sporophyte generation under both sub‐optimum cold and warm temperatures (Gauci et al. 2022). These findings support the potential for thermal priming to improve the thermal resilience of kelps and other marine macrophytes (Jueterbock et al. 2021). The results from this study support these prior observations and, moreover, elucidate the transcriptomic processes underlying the enhanced thermal tolerance observed in primed kelp sporophytes.

### 4.1 Priming Alters Timing and Magnitude of Transcriptomic Responses to Heat Stress

In the heat stress (HT) phase of the experiment, both primed and naïve samples displayed transcriptome changes, with DEGs in both groups clearly indicative of a stress response (Figure 4A). Primed samples showed substantially more DEGs than naïve samples when each was compared to the corresponding starting condition (Figure 4A). The greater number of DEGs in primed samples suggests that priming may enhance the ability of kelps to respond more rapidly or strongly to heat stress, potentially increasing heat resilience. During the recovery phase, naïve samples showed a large number of downregulated DEGs in comparison to the starting point (1397 non‐shared with the primed group, Figure 4A), a pattern not reflected in the primed samples. Comparisons of primed with naïve samples revealed more DEGs during recovery (608) than during heat stress (87, Figure 4B). Overall, GO enrichment patterns in both naïve and primed samples indicate activation of a general stress response, as reflected by terms such as “response to heat” and other stress‐associated processes. This suggests either a later transcriptomic heat stress response or a more severe state of stress in the naïve group.

### 4.2 Differential Activation of Chaperone, Oxidative Stress, and Cell Wall Remodeling Pathways

In addition to enriched GO terms, we observed changes in stress‐related genes (Figure 5 and Data S1), with primed sporophytes mounting a stronger transcriptional response during heat exposure and naïve sporophytes showing more extensive downregulation at the R time point. The predominant downregulation of photosynthesis‐related genes during heat stress mirrors previous findings in *S. latissima* (Heinrich et al. 2012; Nehr et al. 2025) and is consistent with a stress‐induced reduction of photosynthetic activity. The pattern of HSPs, ubiquitins, and oxidative stress‐related transcripts such as thioredoxins, peroxidases, and oxidases broadly agrees with earlier *Saccharina* stress studies (Heinrich et al. 2012; Lin et al. 2024), indicating that both primed and naïve sporophytes engage known heat‐stress pathways but the timing and magnitude of these responses differ between groups. Heat shock proteins, which act as molecular chaperones stabilizing and refolding damaged proteins under abiotic stress (Feder and Hofmann 1999), were strongly represented, and multiple HSP families are known to respond to heat in juvenile *Saccharina* sporophytes (HSP20, HSP40, HSP70, HSP90; Lin et al. 2024). Notably, HSP90B was uniquely upregulated in primed sporophytes at the HT time point, consistent with a qualitatively different chaperone response between primed and naïve groups that may contribute to more effective heat protection in primed samples. The reduced number of HSP‐related DEGs in primed sporophytes at the R time point, relative to naïve sporophytes, supports the interpretation that primed sporophytes had progressed further towards a fully recovered state.

Beyond these gene‐level patterns, GO‐term enrichment further supports a differential stress state between primed and naïve sporophytes at the recovery time point. Genes associated with protein phosphorylation (GO:0006468) showed higher expression in primed than in naïve sporophytes at recovery, and downregulation of protein phosphorylation has been linked to various abiotic stresses in plants such as oil palm (Lee et al. 2024). The lower expression of protein phosphorylation–related genes in naïve sporophytes at recovery therefore suggests that they remained under stronger stress than primed sporophytes. In addition, the GO term post‐transcriptional gene silencing by regulatory ncRNA (GO:1900368) was also enriched in primed sporophytes at recovery. These patterns are consistent with a more active regulatory capacity in primed samples under stress, potentially contributing to enhanced resilience.

An earlier study has shown that *S. latissima* sporophytes characterized as heat stress tolerant or resilient according to their photosynthetic performance in a heat stress experiment (21°C for 7 days) displayed stronger transcriptomic responses to heat compared to sporophytes characterized as sensitive (Nehr et al. 2025). Heat stress tolerant or resilient individuals either maintained high photosynthetic efficiency throughout stress and recovery or recovered within 16 days, respectively (Nehr et al. 2025). QTL mapping has revealed quantitative trait loci (QTL) associated with temperature stress recovery, highlighting the role of genetics in heat stress resilience. Two DEGs from our study (IDs: SL_06_18.3 and SL_06_18.9), both described as pectin lyase‐like transcripts, overlap with these temperature resilience associated QTLs, as upregulated in primed samples in the HT vs. S (SL_06_18.3) and HT vs. R (both transcripts) comparisons, and upregulated (SL_06_18.3) and downregulated (SL_06_18.9) DEGs in resilient kelps under heat stress in the work of Nehr et al. 2025. Thus, our results are in support of pectin‐mediated cell wall remodeling as a candidate pathway for heat stress resilience in *S. latissima*. The direction of regulation and the precise functional role of these transcripts in heat stress responses remain to be clarified in future studies. Our findings that primed samples showed stronger transcriptomic responses during the heat stress compared with the starting point support the idea that priming could complement genetic selection in kelp cultivation by contributing to temperature resilience.

### 4.3 Transcriptomic and Photosynthetic Data Reveal Differences in Stress Responses Between Primed and Naïve Sporophytes

PAM measurements and transcriptomic data together represent different but mutually informative aspects of how primed and naïve sporophytes respond to and recover from thermal stress, with transcriptomic patterns suggesting that primed sporophytes had mitigated stress to a greater extent than naïve sporophytes at the end of the experiment. PAM measurements showed relatively low values (~0.5) for *F*_v_/*F*_m_ before the heat stress compared with the typically observed healthy PAM values for kelps (~0.7) (Dring et al. 1996; Hanelt 1998). The low values may be explained by the developmental stage, as young sporophytes have been reported to show lower *F*_v_/*F*_m_ values than mature kelps (Dring et al. 1996). The primed samples, which showed a stronger transcriptomic response to heat, showed stable photosynthetic performance at the heat stress phase. At the recovery stage only the performance index, and not the quantum yield, was significantly reduced (Data S3 and Figure 2). In contrast, naïve samples showed a significant reduction in both performance index *PI*_*abs*_ and quantum yield from the start of the experiment to the HT phase (Figure 2). Despite the environment having returned to an optimal temperature (10°C) at the recovery time point, neither group had recovered physiologically, as indicated by reduced photosynthetic performance (Figure 2). However, we note that PAM‐derived $F_{v}/F_{m}$ values alone do not fully capture the physiological state of the algae, and our conclusion that primed and naïve samples differed in their recovery is therefore based on the more detailed information provided by the transcriptome profiles. Consistent with this, genes associated with chloroplast functions (GO:0009507) showed enrichment in primed samples compared with naïve at the recovery time point, which may indicate an increased capacity of primed sporophytes to compensate for or repair chloroplast damage, as chloroplasts are particularly susceptible to heat stress (Hu et al. 2020; Mathur et al. 2014). This chloroplast‐related enrichment correlates with the more stable *F*_v_/*F*_m_ values observed in primed sporophytes and supports the interpretation that they had alleviated heat‐induced damage more effectively than naïve sporophytes by the end of the experiment.

In line with these patterns, redox‐related transcripts showed distinct transcriptional differences in primed versus naïve sporophytes. Thioredoxin and ferredoxin–NADP(H) reductase systems are known to play central roles in regulating photosynthetic redox balance and photoprotection under stress in plants (Lodeyro et al. 2021; Nikkanen and Rintamäki 2019; Santos and Rey 2006). In our study, transcripts annotated as thioredoxin‐like and ferredoxin–NADP(H) reductase–like showed an earlier onset of mixed up‐ and downregulation in primed sporophytes, correlating with their slight and delayed reduction of photosynthetic performance during heat stress compared with naïve samples. Together, these findings suggest that a more rapid transcriptional adjustment in primed samples may be associated with the observed effects on photosynthetic performance during heat exposure. Future studies should test this hypothesis by extending the recovery period to later time points and by including growth monitoring or additional physiological stress indicators such as reactive oxygen species to provide a more complete picture of the physiological trajectories of primed and naïve sporophytes.

### 4.4 The Priming Memory Persists From Gametophyte to Sporophyte

The priming treatment, establishing and maintaining a priming memory, can involve trade‐offs such as reduced growth under benign conditions (Hilker et al. 2016; Martinez‐Medina et al. 2016; van Hulten et al. 2006). In this study, the primed samples showed a marginally, though not significantly (*p* = 0.19, Data S3) lower *F*_v_/*F*_m_ than naïve samples before the heat exposure, potentially a cost of the priming treatment. The priming cost may potentially be offset by enhanced resilience to heat stress (Hilker et al. 2016; Martinez‐Medina et al. 2016).

The differential heat stress response of primed and naïve kelp sporophytes on our study suggests a transgenerational priming effect from the gametophyte to the sporophyte stage. However, it is important to note that the transition from gametophyte to sporophyte in the kelp's diplohaplontic life cycle does not involve a meiotic division because meiosis occurs at the transition from the sporophyte to the gametophyte (i.e., during spore production). In consequence, this transgenerational effect is not directly comparable to a generation change involving meiosis, where epigenetic marks may be partially reset through processes such as DNA demethylation (Saze and Kakutani 2007; Kawashima and Berger 2014). Priming of gametophytes may be a unique case, occurring after meiosis and gametophyte growth but before fertilization and the epigenetic reprogramming described during embryogenesis in plants (Kawashima and Berger 2014). This timing could potentially make transgenerational priming more effective than in scenarios where the memory must persist through meiosis. The persistence of the priming memory through fertilization and early sporophyte development indicates that the priming effect goes beyond short‐term acclimation and may involve partially heritable epigenetic mechanisms. The mechanisms of epigenetic inheritance in brown algae, however, are not yet fully understood (Bourdareau et al. 2021) and require further investigation.

The observed priming memory may be explained by a range of epigenetic mechanisms. In this study, primed samples at the recovery time point showed enrichment of chromatin‐ and regulatory ncRNA related GO terms, including “pericentric heterochromatin”(GO:0005721) and the regulatory ncRNA related GO:1900368 (mentioned above). In addition, several histone‐ and methyltransferase‐related DEGs showed a stronger response in primed samples at the HT time point and were differentially expressed between the primed and naïve groups at the R time point (Data S1). Together, these patterns provide preliminary transcriptional support for the hypothesis that chromatin‐ and ncRNA‐based mechanisms underlie priming memory. Non‐coding RNAs, including long non‐coding RNAs (lncRNA), play an important role in the modulation of gene expression and post‐transcriptional regulation during temperature stress in plants (Jha et al. 2020), and may regulate the trade‐off between abiotic stress responses and growth (Zhang et al. 2022). Regulation by ncRNAs has also been suggested as a possible mechanism for the transfer of transgenerational stress memories (Farkas and Dobránszki 2024; Zhang et al. 2022), consistent with the GO:1900368 enrichment observed in this study. DNA methylation is another potential epigenetic mechanism underlying transgenerational stress memory (Farkas and Dobránszki 2024). Although overall DNA methylation levels in *S. latissima* are low and well‐known de novo methyltransferases are absent, its methylome does respond to heat stress and cultivation temperature (Liu et al. 2023; Khatei et al. 2026; Scheschonk et al. 2023, 2024). Regulation of gene expression by DNA methylation is well established, typically involving repression via promoter methylation (Kumar and Mohapatra 2021; Zhang et al. 2018), while gene body methylation may correlate positively with expression, as shown in *Saccharina japonica* (Fan et al. 2020; Liu et al. 2023). Altered chromatin states, including histone methylation marks, are also known to respond to heat stress in plants and may contribute to stress memory (Liu et al. 2022). The observed enrichment of chromatin‐associated GO terms and histone‐related DEGs in our dataset is consistent with these mechanisms and suggests that chromatin state and histone modifications may contribute to stress memory in *S. latissima*. Future studies should explore how epigenetic mechanisms such as DNA methylation, ncRNA, and chromatin and histone modifications correlate with transcriptomic shifts and clarify whether changes to these marks resulting from priming are associated with gene expression changes.

## 5 Conclusion

This study showed that gametophyte thermal priming induces distinct transcriptomic and physiological responses in juvenile *Saccharina latissima* sporophytes under short‐term sub‐lethal heat stress. The transcriptome showed a clear priming effect. Primed sporophytes initiated an earlier and stronger response of genes in stress‐relevant categories, including oxidative stress, photosynthesis, and heat shock pathways, showing unique upregulation of HSP90B, faster regulation of thioredoxin‐like and ferredoxin–NADP(H) reductase–like transcripts, and an early strong downregulation of a specific HSP40/DnaJ transcript, together indicating an altered redox and chaperone response. Primed sporophytes also showed differential regulation of histone and methyltransferase‐related transcripts, consistent with a contribution of chromatin modifications to the priming memory. These molecular differences align with the slight and delayed reduction in photosynthetic efficiency observed in primed sporophytes, supporting earlier studies on the functional benefit of temperature priming. In addition, the differences in the expression patterns of HSPs, and the observed GO enrichments at recovery, suggest that primed sporophytes show improved recovery after thermal stress. Our findings therefore reinforce the idea that gametophyte thermal priming establishes a trans‐generational molecular memory that enhances the speed and effectiveness of stress responses in the sporophyte stage. Future research should explore the specific roles of ncRNAs, histone modifications, and DNA methylation in molecular memories, as well as their correlation with gene expression.

From an applied perspective, our findings on the molecular and physiological effects of priming support its potential for use in kelp aquaculture without compromising genetic diversity. Transcriptomic marks found in this study, such as HSP90B upregulation and rapid transcriptomic response of a range of, for example, ferredoxins and thioredoxins, could be explored as potential biomarkers of successful temperature priming. Optimizing priming methods and combining them with selective breeding could enhance thermal tolerance, improving yield and production security under a warming climate and marine heatwaves. While priming shows a positive effect on the short‐term thermal tolerance of young sporophytes, the duration of this effect and its ability to pass through meiosis remain to be tested. Its potential to mitigate long‐term heat stress for restoration purposes also warrants further investigation.

## Funding

This work was supported by the Research Council of Norway under the KELPRIME project (project no. 700933).

## Conflicts of Interest

The authors declare no conflicts of interest.

## Supporting information


**Data S1:** Differentially expressed genes (DEGs) for all pairwise comparisons, with summaries of DEG counts (Sheets 1.1–1.2) and full DEG lists for naïve Recovery vs. Start (Sheet 1.3), naïve High Temperature vs. Start (Sheet 1.4), primed Recovery vs. Start (Sheet 1.5), primed High Temperature vs. Start (Sheet 1.6), and primed vs. naïve at Start (Sheet 1.7), Recovery (Sheet 1.8), and High Temperature (Sheet 1.9), each annotated with functional information and relevant keywords.

**Data S2:** Gene ontology (GO) enrichment results for differentially expressed genes (DEGs), including enriched GO terms associated with downregulated and upregulated genes in naïve samples at High Temperature vs. Start (Sheets 2.1–2.2) and Recovery vs. Start (Sheets 2.3–2.4), primed samples at High Temperature vs. Start (Sheets 2.5–2.6) and Recovery vs. Start (Sheets 2.7–2.8), and primed vs. naïve samples at the Start, Recovery, and High Temperature time points (Sheets 2.9–2.14).

**Data S3:** Results of repeated‐measures ANOVA and Tukey post hoc tests for photosynthetic performance metrics, including $F_{v}/F_{m}$ and $PI_{\mathrm{abs}}$, presented as repeated measures ANOVA tables (Sheets 3.1 and 3.3) and corresponding Tukey post hoc comparisons (Sheets 3.2 and 3.4).

## Data Availability

The data that support the findings of this study are openly available in The European Nucleotide Archive at https://www.ebi.ac.uk/ena/browser/, reference number PRJEB83977. Read counts, transcript count tables, sample metadata, and R scripts used for the analyses are available from Zenodo at 10.5281/zenodo.20557333.
